# Low MRSA prevalence in horses at farm level

**DOI:** 10.1186/1746-6148-8-213

**Published:** 2012-11-07

**Authors:** Annelies Van den Eede, Ann Martens, Isabelle Feryn, Wannes Vanderhaeghen, Urszula Lipinska, Frank Gasthuys, Patrick Butaye, Freddy Haesebrouck, Katleen Hermans

**Affiliations:** 1 Department of Surgery and Anaesthesiology of Domestic Animals, Faculty of Veterinary Medicine, Ghent University, Salisburylaan 133, 9820, Merelbeke, Belgium; 2 Veterinary and Agrochemical Research Centre, CODA-CERVA-VAR, Groeselenberg 99, 1180, Ukkel, Belgium; 3 Department of Pathology, Bacteriology and Avian Diseases, Faculty of Veterinary Medicine, Ghent University, Salisburylaan 133, 9820, Merelbeke, Belgium

**Keywords:** Horse, *Staphylococcus aureus*, MRSA, Methicillin resistance, Horse farm

## Abstract

**Background:**

In Europe, methicillin-resistant *Staphylococcus aureus* (MRSA) belonging to the clonal complex (CC) 398 has become an important pathogen in horses, circulating in equine clinics and causing both colonization and infection. Whether equine MRSA is bound to hospitals or can also circulate in the general horse population is currently unknown. This study, therefore, reports the nasal and perianal MRSA screening of 189 horses on 10 farms in a suspected high prevalence region (East- and West-Flanders, Belgium).

**Results:**

Only one horse (0.53%) from one farm (10%) tested positive in the nose. It carried a *spa* type t011-SCC*mec*V isolate, resistant to β-lactams and tetracycline, which is typical for livestock-associated MRSA CC398.

**Conclusion:**

In the region tested here, horses on horse farms seem unlikely to substantially contribute to the large animal associated ST398 MRSA reservoir present at intensive animal production units.

## Background

Since its first discovery in horses in 1989 [1], methicillin-resistant *Staphylococcus aureus* (MRSA) has clearly emerged as an important pathogen in equine clinics, causing both carriage (colonization) and infections in patients and personnel [1-8]. Whether MRSA is also circulating in the equine population outside clinics, as in humans and livestock [9,10], is however much less known. Such carriage in the general population could be very important since colonized horses are at an increased risk of developing infections themselves and may spread the pathogen to other horses, animal species and their human caretakers [3,6,11]. Current literature on the presence and extent of an equine MRSA reservoir at farm level is rather limited and demonstrates regional differences in carriage rates (0-4.7%) and strain types [12-16], a phenomenon typical for MRSA carriage [17-19]. On the European mainland, the colonization status of healthy horses outside clinics is largely unknown, with only two studies reporting on seemingly negative populations [13,14].

At the beginning of the 2000s, MRSA belonging to the clonal complex (CC) 398 was isolated from pigs and pig farmers in the Netherlands [20]. These so called livestock-associated-MRSA (LA-MRSA) strains are highly prevalent in pigs in European countries [9]. Although pigs act as the main reservoir, LA-MRSA CC398 has also been shown to be present in other food producing animals, including poultry [21,22] and cattle [23], and it has occasionally been detected in companion animals [24]. In horses, LA-MRSA CC398 is currently regularly being detected in European equine clinics with up to 55% of hospitalized animals testing positive [5-9,25,26].

In a previous study, 10.9% of the horses arriving at an equine clinic in Flanders, Belgium, carried LA-MRSA CC398 in their nose [6]. Although it has been suggested that these horses might be representatives of the general population [6,7], it cannot be excluded that patients arriving at a clinic represent a biased subpopulation. It was therefore the aim of the present study to assess if LA-MRSA CC398 is really endemically present in the general horse population, by screening horses on horse farms for the occurrence of MRSA.

## Results

The screened horses were between 3 and 26 years of age (mean age 8.3 years with standard error of this mean of 0.4 years) with 73 (38.6%) of them being male [19 (10.1%) stallions, 54 (28.6%) geldings] and 116 (61.4%) being female. The sampling population existed of 120 (63.5%) warmbloods, 21 (11.1%) standardbreds, 22 (11.6%) draft horses (Brabant, Haflinger, Fjord), 9 (4.8%) thoroughbreds (Arabian, Anglo-Arabian) and 14 (7.4%) others (other breeds and crossbreds).

In total, 373 samples (188 nasal swabs + 185 perianal swabs) from horses were gathered. From one horse no nasal swab and from four other horses, no perianal swab could be taken due to signs of resistance in the animals.

From one nasal swab, a MRSA isolate was obtained with a *spa* type t011 strain carrying a SCC*mec*V cassette. MRSA was not isolated from any of the other samples. Phenotypically, the obtained MRSA isolate showed acquired resistance to oxacillin and tetracycline.

The animal from which the MRSA isolate was obtained was a warmblood, breeding mare, housed at a breeding facility with about 70 horses.

## Discussion

In the present study, only 1 out of 189 horses from 10 different farms carried a typical representative of the LA-MRSA CC398 clone. The farms were all situated in a region surrounding an equine clinic where shortly before, high carriage rates had been detected in arriving patients [6]. The fact that such a low presence of MRSA on farms was found in this study is rather surprising, and in contrast with the earlier described high carriage rate in horses arriving at the clinic. Possible factors responsible for the higher carriership in equine patients admitted to a clinic may be previous antimicrobial administration, stress due to transportation, transport in contaminated vehicles and direct contact with referring veterinarians who might carry MRSA. Large animal veterinarians are indeed considered to be at high risk of carrying the strains circulating in their main contact species [27-30].

The finding that only one horse was found to be MRSA positive was all the more unexpected since the samples were collected on rather large (n > 20) horse farms, which are more likely to harbor MRSA carriers [12] and the study was performed in a region where the LA-MRSA CC398 clone is highly prevalent in pigs, with high carriage rates detected at human, individual pig and farm level [31,32]. Although contact of the horses in the current study with pigs was not assessed as such, it would not have been impossible for pig to horse transmission of CC398 to occur, especially given the long term environmental survival, potential airborne transmission of MRSA and the possibility of large animal veterinarians circulating between pig and horse farms [33-36]. In the current study, the only positive horse was a breeding mare housed on a breeding facility with consequent regular veterinary contact and a high number of horses present in the facility.

The low MRSA presence in the general horse population found here is in accordance with recent findings in the Netherlands where the detection of high CC398 prevalences in livestock (pig, veal calves, poultry), equine clinics and veterinarians [7,37-40] also stands opposite to a study detecting no MRSA in the general horse population [13]. Both Dutch and Belgian data thus seem to classify equine CC398 carriage as a primarily veterinary-care associated problem with high detection rates only being found in horses arriving and residing at veterinary clinics. This difference in MRSA prevalence found between horse farms and intensive food production animal facilities seems unlikely to be due to differences in host adaptation given the substantial MRSA carriage found in equine clinics [7,26]. It could, however, be due to differences in husbandry between the examined horse farms and intensive animal husbandry in the same region. For instance, the animal density on horse farms is much lower and group medication (antimicrobials) is virtually non-existing. In fact, the conditions encountered in equine clinics concur much more with those in intensive animal production and may thus, partially, explain the high MRSA prevalence encountered in equine clinics. Common factors in the housing and husbandry of high prevalence sectors in the animal industry should probably be the first to scrutinize when examining potential risk factors for animal LA-MRSA carriage. In addition to the nature of equine husbandry, a second factor may counteract the spread of LA-MRSA in the general horse population. Indeed, horses appear to be mainly transient carriers eliminating MRSA quickly (< 3 weeks) when removed from potential contamination sources [41]. If contaminated at clinic, they may thus quickly eliminate the bacterium when at home.

## Conclusion

In conclusion, our results indicate that the prevalence of equine LA-MRSA is low on horse farms. Continued monitoring is, however, advisable to avoid missing the potential future emergence of MRSA in the general, healthy horse population.

## Methods

### Study population and sample collection

The study was conducted in accordance with the Belgian Law of 14 August 1986 and the European Directive 86/609/EEC.

A total of 189 horses from 10 farms (15 to 21 animals per farm) housing at least 20 horses and/or ponies, were screened between January and March 2008. The farms were all situated in the provinces of East- and West-Flanders (Belgium), in a region surrounding an equine clinic where five to nine months before, high carriage rates (10.9%) had been detected in arriving patients. Animals were chosen according to availability for immediate sampling and owner compliance.

All animals were swabbed both in the nose and at the perianal region using cotton-tipped swabs embedded in solid Stuart’s medium (UNI-TER AMIES CLR, Piove di Sacco, Italy), as previously described [2,3,6,26]. Samples were held at 4°C for a maximum of 24 hours before transfer to the lab.

### MRSA isolation, typing and antimicrobial susceptibility testing

Samples were cultured according to Van den Eede et al. [6] using a 0.001% colistin and nalidixic acid containing enrichment broth and chromogenic MRSA screening agar (ChromID^TM^ MRSA, bioMérieux, Lyon, France) with isolation and further phenotypic identification (colony morphology, haemolytic capacity, DNAse and catalase activity and growth on a modified Baird-parker agar). A previously developed duplex PCR [6], using the primers described by Mehrotra et al. [42] was used to confirm *femA* (*S. aureus* specific) and *mecA* (methicillin resistance) gene presence in MRSA suspected isolates. Positive isolates were further characterized by *spa* typing [43] and SCC*mec* typing [44,45].

Antimicrobial susceptibility to tetracycline, enrofloxacin, erythromycin, tylosin, clindamycin, lincomycin, sulfonamide, trimethoprim, gentamicin and neomycin of MRSA isolates was determined using the Kirby-Bauer disk diffusion method, with Neo-Sensitabs^TM^ (Rosco Diagnostica A/S, Taastrup, Denmark) on Iso-Sensitest agars (Oxoid, Wesel, Germany), incubated at 35°C. Inoculum standardization, medium and incubation conditions as well as interpretation of inhibition zones were performed according to the tablet manufacturer’s guidelines (Guidelines for the use of Neosensitabs^TM^, 18^th^ ed., 2005/2006, http://www.rosco.dk). Oxacillin resistance was similarly tested but with incubation at 30°C for 48 hrs.

## Abbreviations

CC: Clonal Complex; LA-MRSA: Livestock-Associated Methicillin Resistant *Staphylococcus aureus*; MRSA: Methicillin Resistant *Staphylococcus aureus*.

## Competing interests

The authors state that there is no conflict of interest with regard to this study.

## Authors’ contributions

AV was the PhD student who conducted the study, together with IF. KH and AM designed the study and were direct supervisors. UL and WV performed molecular typing. FH, PB and FG contributed to interpretation of results and to the design of the final manuscript. The manuscript was prepared by AV and corrected by all other authors. All authors read and approved the final manuscript.

## Authors’ information

Freddy Haesebrouck and Katleen Hermans, Department of Pathology, Bacteriology and Avian Diseases, Faculty of Veterinary Medicine, Ghent University, Salisburylaan 133, 9820 Merelbeke, Belgium.

Freddy Haesebrouck and Katleen Hermans shared senior authorship.
